# “Being brave, being seen, and having your voice heard”: Perspectives of self‐advocates and families toward accessible and impactful research of Alzheimer's disease in down syndrome

**DOI:** 10.1002/alz.70999

**Published:** 2025-12-19

**Authors:** Sarah Walter, Lauren Ptomey, Elizabeth Head, Ann Cohen, Joseph Mike Briones, Henry Shaw, Brandon Carter, Jessica Kishner, Willie Pestolesi, Anthony Sciullo, Katy Kolb Olmstead, Aurelia Carter, Sara Kishner, Amy Kolb Tucker, Lissa Pestolesi, Maria Briones, Dana Sciullo, Kay Sciullo, Pam Shaw, Perry Chen, Michael S. Rafii

**Affiliations:** ^1^ Alzheimer's Therapeutic Research Institute University of Southern California San Diego California USA; ^2^ Department of Internal Medicine The University of Kansas Medical Center Kansas City Kansas USA; ^3^ Department of Pathology & Laboratory Medicine, and Neurology School of Medicine University of California, Irvine Irvine California USA; ^4^ Alzheimer's Disease Research Center University Of Pittsburgh School of Medicine Pittsburgh Pennsylvania USA; ^5^ Alzheimer's Therapeutic Research Institute, University of Southern California Alzheimer's Clinical Trials Consortium Down Syndrome and Alzheimer's Biomarker Consortium Down Syndrome Research Partnership Group San Diego California USA

**Keywords:** Alzheimer's disease, biomarkers, clinical trials, community advisory board, community engagement, Down syndrome, genetic risk, intellectual disability, lived experience, observational studies, partnership, return of results, underserved communities

## Abstract

**INTRODUCTION:**

Over 220,000 people live with Down syndrome (DS) in the United States, with an average life expectancy of 63.5 years of age. The high risk for Alzheimer's disease (AD) in DS means studies must be accessible and inclusive. We share our experience of building a collaborative group of self‐advocates and care partners to provide meaningful feedback on research.

**METHODS:**

Our research partnership group is supported by the Alzheimer's Clinical Trials Consortium—Down Syndrome (ACTC‐DS) and Alzheimer's Biomarker Consortium Down Syndrome (ABC‐DS). We synthesize feedback into themes and recommendations for researchers.

**RESULTS:**

Feedback themes are broad, extending from the strong motivation to engage in research to the need to balance study burden and risk. Recommendations for researchers include providing support and connection to resources, sharing individual and study‐level results, flexibility, and respectful communication.

**DISCUSSION:**

Our collaborative approach will lead to more relevant and accessible research studies.

**Highlights:**

- Inclusive and accessible research is needed for Down syndrome and Alzheimer's disease.
- We built a collaborative group of self‐advocates and care partners to provide feedback.
- Feedback includes a strong motivation to learn about brain health and engage in research.
- Respectful communication includes representative images and tailored materials.
- Recommendations include the choice to learn individual results, flexibility, and support.

## 1 BACKGROUND

Over 220,000 people live with Down syndrome (DS) in the United States. Advances in healthcare and support have contributed to improved average life expectancy of 63.5 years of age.^1^ Individuals with DS carry a high risk for dementia caused by Alzheimer's disease (AD), due to the triplication of chromosome 21, which leads to the overexpression of the amyloid precursor protein (APP) and amyloid deposition in brain.^2^ Other contributors in DS include the high level of tau tangles, neuroinflammation and oxidative stress, and vascular impairment.^3^ AD is now the leading cause of death for individuals with DS over the age of 35 years, with an average age of diagnosis around 55 years.^4^ Given the genetic basis for Alzheimer's disease in Down syndrome (DSAD), DS is considered stage 0 on the Alzheimer's disease continuum—a spectrum that begins with elevated risk (including DS) and progresses through to the impaired stage of dementia.^5^ Caregiver research reveals that the top concerns after the age of 40 years are around neurological issues and accessing specialty care.^6^ Research in sporadic AD has led to two disease‐modifying therapeutics approved by the United States Food and Drug Administration (FDA) and over 180 clinical trials testing additional interventions.^7^ These advances offer promise in the face of what was previously a hopeless diagnosis. Unfortunately, people with DS have largely been excluded from engaging in research that informs their health, including dementia research, despite strong interest from the community.^8^ The Alzheimer's Clinical Trials Consortium Down Syndrome (ACTC‐DS) was established to accelerate the development and conduct of clinical trials.^9^ To bridge the gap in research, the US government funded an initiative across the National Institutes of Health (NIH) called Investigation of Cooccurring Conditions across the Lifespan to Understand Down Syndrome (INCLUDE). INCLUDE has led to increasing numbers of studies specifically related to improving the health of individuals with DS.^10^ The NIH INCLUDE initiative assembles leading clinicians, scientists, and representatives of advocacy groups to review existing methods and to identify those areas where new approaches are needed to engage and prepare populations with DS for participation in clinical research. Subsequently, a roadmap was developed by a working group on conducting clinical trials in persons with DS including best practices for engaging this community.^11^

Self‐advocates express a strong desire and capability to engage as participants, as coresearchers, and provide guidance on research study design and conduct.^12^, ^13^ Public and participant involvement (PPI) is a tool for inclusive dementia research, particularly critical to reach minoritized and underserved communities.^14^ Patient Advisory boards that facilitate ongoing feedback from individuals with lived experience improves study design and participant experience. We recognize the need for ongoing feedback and guidance from self‐advocates as well as family members.

Integrating the voices of those with lived experience is essential in both observational and interventional DS research. Close partnership ensures ethical integrity and meaningful, impactful, and inclusive studies, for a group that has long been excluded from research.^12^ Individuals with DS and their families offer insights and priorities that researchers alone cannot fully anticipate. Their involvement enhances study design, outcome selection, and recruitment and retention strategies, aligning research more closely with the needs of the community it serves.

In observational studies, participant input helps shape research questions around real‐world concerns, such as daily functioning, social inclusion, and cognitive change, ensuring that collected data reflects lived realities and translates into actionable improvements. In clinical trials, engagement fosters trust, improves recruitment and retention, and ensures that interventions are tested in ways that respect the dignity and autonomy of participants. Partnership with lived experience improves communication, promotes independence and quality of life, and outcomes are more relevant and meaningful.

We have assembled a national Down syndrome Research Partnership Group to support on‐going and upcoming observational studies and clinical trials. In this paper we aim to: [a] share our experience of building a collaborative group to provide meaningful feedback on research and [b] share initial impact and key themes of feedback discussed by our group. We have followed guidance for meaningful PPI by including our group members as coauthors. To ensure accessible translation of our learnings to community members, we have also cocreated a version of this paper that is accessible for people with DS in Supporting Information.

## 2 METHODS

Our Research Partnership Group was formed in 2024, with support from two NIA‐funded research consortia: The ACTC‐DS and Alzheimer's Biomarker Consortium Down Syndrome (ABC‐DS).^3^

The principles of collaboration and partnership guide our approach. We prioritize building personal relationships and sharing lived experiences, following previously published recommendations, including the Patient‐Centered Outcome Research Institute (PCORI) principles^15^ and methods for building meaningful PPI across the research lifespan.^14^ Key strategies we employ include: regular meetings, flexibility in scheduling at times that are best for self‐advocates and family members, asking group members for topics they would like to discuss, providing materials and discussion questions at least 2 weeks in advance, intentionally structuring meetings to ensure self‐advocates speak first, creating space for group members to speak or listen based on their comfort, and time to socialize and connect. Researchers in the group set the tone for open dialogue by focusing on listening, and demonstrating gratitude for feedback, especially when members are sharing difficult experiences or identifying a “blind spot” of researchers. Offering flexibility like (one one‐on‐one follow‐up), and providing meeting summaries, as well as sharing the impact of the group's feedback is also key.

Group candidates are identified by Down syndrome researchers involved in ABC‐DS and ACTC‐DS studies and are given a one‐page description of the group purpose and role of members. Members are recruited in dyads, including the person with DS and at least one care partner. Potential members are not required to have experience in research, but must be willing to share their perspective. If potential members are willing to learn more, a zoom meeting is set up with the primary group facilitator (S.W.) to discuss what involvement would look like. We aim to balance small group size to promote relationship building, while ensuring representation of varied research experiences, geography, age, race, and ethnicity. Zoom meetings occur every 2 months for 1 hour, and topics are generated by both group members and researchers. Zoom meetings include both the self‐advocate and care partner, and communication and scheduling are primarily through emails or text messages with the care partner. One researcher with experience in facilitating advisory groups (S.W.) leads group meetings and facilitates discussion and feedback. The other researchers have different levels of experience with engaging with community members, and participate in group meetings primarily in a listening role. Researchers will present updates when a topic is requested by group members. For example, group members have asked for updates on clinical trials in DS, exercise recommendations, and strategies for aging well with DS. Additionally, in‐person meetings occur once a year, in conjunction with national DS conferences, with informal gatherings throughout the year around other meetings and local events. Our first in‐person meeting focused on capacity building for both self‐advocates and care partners. In‐person meetings include separate discussion groups for care partners and self‐advocates to build group cohesion, confidence, and to develop shared priorities and goals.

**RESEARCH IN CONTEXT**

1. **Systematic review**: There is very limited research on how to design inclusive and accessible research for Alzheimer's disease in Down syndrome (DS). Our group of self‐advocates and care partners generated feedback and recommendations.
2. **Interpretation**: The feedback provided includes experiences of discrimination in health care, and the need for respectful communication and support in research. Recommendations for researchers include the option to learn individual research results, provide updates to communities, flexibility in study procedures, and connection to clinical care and social support.
3. **Future directions**: We urge researchers to develop meaningful partnerships with individuals with DS and their families. The impact of these collaborations will be research that is relevant and accessible.

The collection and dissemination of feedback from our partnership group is under a research protocol determined to be exempt by the University of Southern California Institutional Review Board. We also collect confidentiality agreements and permission for use of video and photos. Group members are reimbursed at a rate of $50 dollars per hour. We cover travel costs for both the self‐advocate and care partner to attend one DS conference each year. The results in this paper emerged from a review of meeting recordings. A summary of themes and recommendations for researchers were presented to the group members, and their feedback was incorporated.

## 3 RESULTS

### 3.1 Group composition

Seven self‐advocates make up our research partnership group, along with eight care partners and five researchers. Geographically our group includes six self‐advocates and care partners from California, two from New Jersey, five from Pennsylvania, and two from Kansas. Researchers in the group are from University of California, Irvine, University of Pittsburgh, University of Kansas, and University of Southern California. The self‐advocates range in age from 25 to 45 years. One self‐advocate lives with early‐stage cognitive impairment. Two of our self‐advocates are women and five are men. Our initial set of care partners are all women, with six parents and one sibling. Four members self‐identify as African–American and two identify as Latino/Hispanic ethnicity. Self‐advocates and care partners were identified by a researcher from their area either as part of a community advisory board or participating in a current DS study. One parent reached out by email looking for ways to contribute and was subsequently invited to join the group along with her son. Members bring to the group varied experience with research, with some participating in longitudinal studies and some that have never taken part in research.

The group held seven zoom meetings between January 2024 and June 2025, and met at three DS conferences, with one full day meeting in July 2025. Topics have evolved from providing feedback from self‐advocates and care partners on three new clinical trials, to the composition of participant‐facing materials, to addressing member questions about AD, leading to codeveloping educational and decision guides. The following sections share the results of feedback gathered from the Down Syndrome Research Partnership Group, followed by key recommendations for researchers.

### 3.2 Themes

#### 3.2.1 Motivation

Self‐advocates and family members both hold a strong motivation to engage in research. Self‐advocates share how highly they value their independence, their role in their family and community, including their activities and jobs. Every member of the research partnership group has a friend or family member to AD, and three of the self‐advocates have a grandparent currently living with dementia.

One of the self‐advocates on our group who is beginning to experience changes in her cognition shared: *“If something happens to me, I might die. I saw it on Facebook…this is one of the negative things in my head all of the time. I'm scared every day. I don't like this, how I feel every single night. Sometimes I think that's where I belong, I just get scared for my whole life.”* (K.K.O. – self‐advocate) Our group discussions have revealed that although it is difficult to discuss the very high risk for future dementia, it is important to have conversations early so that the self‐advocate and family can plan. *“This is hard, but this is reality—we are not hiding from that.”* (A.K.T. – care partner) *“We weren't talking about Alzheimer's, but we are now.”* (A.C. – care partner)

#### 3.2.2 Balancing risk

Self‐advocates and family members are aware of their medical issues and are concerned when studies exclude conditions that are common in DS (e.g., auto‐immune conditions, diabetes, gluten intolerance, hearing and speech impairments, and hypothyroidism). Some express concerns about contraindications for their existing medications and the investigational product being tested. When evaluating a study as a fit for them, they are balancing their own future risk for dementia while continuing to manage their current health conditions, against the potential risk for short‐ or long‐term side effects. *“Will the research study conflict with my daily schedule and also my daily psoriatic arthritis and psoriasis medication? It goes to other people who actually have same medical problems, what will it mean for them to go through that.”* (J.K. – self‐advocate)

Researchers should be up‐front about what other medical conditions would be of concern for the study, and consent forms should encourage participants to talk about their medical issues, possible side effects, or concerns in detail with the study physician prior to making a decision to join a study. Some self‐advocates in our group assumed the study they were reviewing would not allow their current conditions. To mitigate concerns of potential participants, researchers should make clear in recruitment materials that some medical issues can be accommodated and that a discussion with the study physician will help to answer questions around possible side effects. Study partners in our group stated that study materials must be clear, representative, and accurate; otherwise the study partner did not feel the study team could be trusted with their loved one.

#### 3.2.3 Respect for self‐advocates

Self‐advocates have shared that it is common for medical staff to not speak directly to the person with DS. Group members share stories of being ignored, as well as strategies they use to label the behavior. *“It's a huge battle for me using my words. People think I don't understand, but I do understand. Mostly I do. I just can't find my words, so people don't always talk to me. They talk to you [mom]. Even [mom] sometimes try to answer for me—but I'm not finished, I just can't find my words.”* (K.K.O. – self‐advocate) Another common shared experience was research studies that do not fit within the busy lives of the self‐advocate or their family members. For example, one self‐advocate works full‐time and is not available to participate in research activities during daily work hours. Researchers can demonstrate respect for persons by ensuring maximal flexibility, availability on evenings and weekends to avoid loss of work and disruption to important weekly routines. Respect for self‐advocates also means ensuring comfort with every step of the research visit, taking the time to explain what will happen and working together with the self‐advocate to discuss how they can be comfortable. *“Can I have someone there to support me while going through the study? …I want people to tell me, to talk me through it to, tell me what they are doing and things like that, so I know what they are doing.”* (A.S. – self‐advocate)

Group members describe the sharing of individual results as demonstrating respect for them as individuals: *“I am actually going to find out from all my results, all my PET scans, all my MRI results…”* (W.P. – self‐advocate) Understanding is enhanced by providing examples: *“For my son, I had to give him examples of what possible side effects are, what exactly does that mean? I had to start at that very base level, I used examples of his medications that he is taking now to talk about some of the things that could happen.”* (A.C. – care partner) Finally, group members shared feedback on study communication emphasizing the importance of using images of people with DS from different racial and ethnic backgrounds, with accessible and understandable language that defines all key terms. *“If I don't see someone that looks like me, I assume you don't want me.”* (A.C. – care partner)

#### 3.2.4 Limited information and speciality care

There is a dearth of specialty care or even sufficient information for self‐advocates and families to use to support healthy aging. Research, when it can be accessed, is one strategy that is employed to garner the best medical advice. Benefits include identifying undiagnosed conditions during the screening process for research studies. Self‐advocates want to make informed decisions to reduce their own risk. In our group, we have intentionally created space for open dialogue. Questions raised by our group range from guidance on diet and exercise (e.g. *“What is the right kind of exercise I should be getting, and how much?”* [J.K. – self‐advocate]) through to unanswered questions that require further research like the relationship between menopause and dementia in DS. Our group asks for updates on new research, both from the studies they are involved in as well as others. Families are connected through both formal networks, including the National Down Syndrome Society, National Down Syndrome Congress, and Special Olympics, but also through informal networks, and frequently relay information through these networks to others (e.g., Facebook). Researchers can fill this critical need through regular communication with participants, study partners, and communities through all stages of a research project.

#### 3.2.5 The value of peer support

Self‐advocates in our Research Partnership Group express how important they have become to each other, and the inspiration they draw from the examples of others. *“I know where she comes from, I know she's kind of scared, and I will be beside her.”* (K.K.O. – self‐advocate) *“I did want to say, Thank you for thinking of this for me…I do have to say a huge thank you as well, because I've been very happy to be in a group of people that I know and I actually like them, so this is a really good friendship, so I want to say thank you for that. I was saying about [another self‐advocate], he was answering all of your guy's questions and I was like ‘Wow, he is super confident!’ And when I hear how much confidence each person has, I just think it's brave, and it's really about having your voice heard and to be seen.”* (J.K. – self‐advocate) The value of peer support is best demonstrated by one self‐advocate who initiated and leads monthly “social time” zoom calls for self‐advocates in our group.

### 3.3 Recommendations for researchers

#### 3.3.1 Support and connection

Research staff should provide support and connection to other resources, including research studies if they exist. Screening for research, in particular a clinical trial, is a particularly sensitive phase for people who face extremely high lifetime risk for dementia and a complex medical history, coupled with limited research opportunities and strict criteria for studies. Sharing as much detail up front about study requirements can reduce emotional impact when a participant does not qualify. Many self‐advocates and families turn to research to fill a gap in specialty care and support. Understanding their motivations and connecting them to resources will allow research teams to provide the best support for participants who are not eligible, and potentially invite the participant to consider other studies.

#### 3.3.2 Individual research results

Offer the choice to learn individual research results. One of our care partners shared, after some hesitation, that the reason they decided not to sign up for research was because there was no information that would be shared, in addition to the challenge of a 2‐hour drive to the study site. Another participant who is just now learning his results from a longitudinal study he has been involved in for a few years, shared how important it is for him to see the scans and understand how his brain has changed during his lifetime. The feedback we received confirms that receiving individualized research results can be highly important to both care partners and participants. Offering the choice to access these results may incentivize clinical trial enrollment, support project involvement, and empower participants who are interested in understanding their brain health.

#### 3.3.3 Review research basics

Do not assume that self‐advocates and families are familiar with the research process. Take time to talk about what a research study is, what a placebo is, what is in the placebo, how participants are supported in a study, how safety is monitored, and emphasizing their choice. The questions from our group ranged from asking who will be with them during a study visit, will a procedure be painful, how long with the pain last, can some procedures be done at home or by phone? This pointed to the importance of researchers to both tailor study materials for self‐advocates but not to oversimplify the description of procedures. Researchers should describe each procedure completely including whether the self‐advocate will be alone or with a support person and whether there is pain or discomfort involved. Our experience found that it was only by reviewing draft materials with self‐advocates that we were able to identify what was important to include. Based on this experience, we encourage all researchers to review study materials with self‐advocates and study partners prior to finalization (Table 1).

**TABLE 1:** Recommendations for researchers from self‐sdvocates and care partners.

Recommendations	
Provide connection to resources, social support, clinical care or other studies Offer the choice to learn individual research results, and provide accessible materials to explain what results mean Take time to describe steps involved in research, define key terms, and how safety is tracked Review how participants are supported in a study and promote independence and choice Design research to fit in the lives of self‐advocates and families	Train all staff who interact with participants to speak directly to the self‐advocate, and ask for feedback on communication Discuss with self‐advocates which procedures are difficult and develop a plan for research visits that includes breaks Share research learnings to the Down syndrome community and with participants, including those that did not qualify Partner with self‐advocates as coresearchers and ambassadors for research

#### 3.3.4 Tailor research to busy lives

Ensure that the research can fit into the lives of the self‐advocate and support partner. Self‐advocates may be working either full‐ or part‐time. For those that do not work, schedules may be highly structured throughout the day and evening, which makes it very disruptive and difficult to participate in research. Even where work offers flexibility or is unpaid work, our self‐advocates express high personal value in being reliable to their coworkers, and shared that taking time off is distressing and reduces their pride in their work. Researchers can provide a brief summary of the project that self‐advocates could share with employers, if desired, for explaining why work is being missed. Many support partners for self‐advocates are a parent and both the self‐advocate and care partners may be working, requiring evening and weekend clinic visits and scanning appointments. Driving is a barrier and easily accessible transportation and parking should be provided. Discuss with self‐advocates and families what would be helpful to ease transportation stress. Consider partnering with local hospitals and laboratories, or in‐home services, for some research procedures to minimize travel and impact on the self‐advocate's schedule and on family. For care partners that have flexible work arrangements, providing a quiet place where they can work during the research visits can minimize impact.

#### 3.3.5 Respectful communication

Train all of the research staff who will interact with participants to speak directly to the self‐advocate. Discuss communication preferences up‐front and ask for feedback on your communication style from the self‐advocate and care partner. For example, self‐advocates in our group have expressed how important eye contact is, difficulty in understanding people wearing a mask, or when people speak too fast, use acronyms, and use infantilizing language. Adapt your interviews and forms to support the participant having an active role in the study. This might look like worksheets to help prepare for the research visit, log side effects, or medication use. Review those materials with participants to ensure you have developed something that will be useful. When discussing the study, including visits, procedures, and possible side effects, self‐advocates from our research group have asked for these conversations to be individualized with examples from their life. Our group discussions have revealed a primary concern of self‐advocates is whether someone could be with them at the study visits.

#### 3.3.6 Support during research

Build time into research visits for breaks, especially if a cognitive test or procedure is stressful or uncomfortable. Provide activities while waiting between procedures, allow family members to come into the MRI and PET suite. Ask the self‐advocate their favorite music, video game or other way to relax and decompress so that the research visit is built around what works best for them. Be upfront and discuss with the self‐advocate and study partner which procedures will be painful or uncomfortable. The pain from needles, band aids, and sticky pads for electrocardiograms (EKGs) were all frequently raised by self‐advocates as a concern. Discuss what is most uncomfortable for them so you understand how to best support both the participant and study partner. For study partners, managing anxiety and reactions of the self‐advocate is a significant barrier for taking part in research. Solutions that have been shared by our group include topical use of black pepper essential oil or lidocaine to numb skin prior to blood draws, as well as using oils to minimize pain when removing tape.

#### 3.3.7 Accessible materials with inclusive images

Prepare accessible study materials for self‐advocates to read for themselves and review with family. People with DS deserve clear information that communicates their risk and what can be done to age well in the face of that risk. Study materials should use images and videos of people with DS from diverse backgrounds. Images should reflect what will actually happen in the study, and reflect how the participant is supported during procedures. All scientific terms should be defined. Researchers should reference available resources for best practices to design information materials (e.g. the Center for Disease Control's product development tool for adults in Intellectual Development Disorder). Our group has piloted a codesign process with our group with multiple rounds of writing, revision, and formatting to develop informational materials. We encourage researchers to develop local advisory boards to review participant‐facing materials and ensure all participant‐facing materials are readable, understandable, and relevant to their lives and the community. We note that there are very limited resources with respect to icons/symbols and visual graphics depicting people with DS engaged in research procedures offering an opportunity for future development in collaboration with self‐advocates.

#### 3.3.8 Ongoing connection and research updates

Plan on regular connection with your participants as well as those that don't qualify or haven't signed up for research yet. Show up regularly and you will build the trust that is needed. This type of participation could include engaging in Buddy Walks, local DS Association activities, and creating events that celebrate the abilities and interests of self‐advocates, like University of California at Irvine's Down Syndrome Showcase.^16^ ABC‐DS is implementing an initiative where every research paper includes a lay summary to be shared with community members.

#### 3.3.9 Meaningful partnership

Self‐advocates in our group express eagerness to do more than volunteer as participants, and some have offered to work as coresearchers and as partners in the research process.^15^ Our self‐advocates share that their involvement on the research team provides actionability for those not eligible for studies, much‐needed peer support, and an opportunity to help researchers. Researchers have successfully hired self‐advocates as research staff and coresearchers.^17^ Self‐advocates have authored papers in scientific journals on the importance of research in AD and DS.^12^, ^18^ Our group experience demonstrated that time is required for trust to build, and closer personal relationships yield the most honest feedback, especially around sensitive issues like maintaining independence and losing friends and relatives.

## 4 DISCUSSION

The ACTC‐DS and ABC‐DS research partnership group has provided important feedback on how to more effectively design and conduct AD studies, embedding the voices of individuals with DS and their families into the design and conduct of both observational and intervention research of AD. Early results of this collaboration include revisions to our study protocols, informed consent process and forms, development of accessible informational materials, and strengthened trust between researchers and the DS community. This work builds on earlier calls for greater inclusivity in AD research. Strategies that have yielded success in other under‐served communities should be employed in DS. Researchers should plan to engage openly with the DS community and begin by asking what they can do to meet the needs of the community before asking for engagement in research.^14^, ^19^, ^20^ Our partnership group represents a practical realization of these visions, operationalizing community engagement into tangible changes to the approach of researchers. Their feedback extends from how studies are designed, to implementation, how communication is tailored, through the dissemination of learnings from research.

Reducing study burden allows research to be more accessible for both self‐advocates and care partners. To reduce study burden, it is important to understand what is important to the self‐advocate and how the research can best fit into their lives as individuals and as part of their communities. Flexibility in scheduling visits and allowing for remote assessments and follow‐up will reduce burden and lead to better retention. Previous studies emphasize the importance of pre‐education and minimizing study‐related risks,^21^ which is substantiated by the feedback we have received from our group.

The combination of the 90% lifetime risk for dementia in people with DS and limited access to health care means it critical for researchers to ensure that there is no research without care and connection to support services.

Our group's feedback confirms the importance of disseminating research learnings to the DS community, to empower self‐advocates and care partners to make the best possible decisions about their health and to plan for aging. We learned that families are also a conduit through which research learnings can flow to primary care providers and specialists like neurologists. Sharing results of research can build trust and hope for better health outcomes for all individuals with DS and could galvanize participation in research.

Our experience in building this partnership group has put to rest myths that have limited research opportunities for this important population: (a) people with DS have the interest and enthusiasm to engage in research, including both observational and clinical trials; and (b) people with DS can make decisions about their health and want to understand how to age well, including having tough conversations about their risk for AD.

Building opportunities for peer support and connection is an important part of our group's process, contributing to stronger trust with each other and more in‐depth discussions as well as honest feedback on sensitive topics, which may not have been possible without the relationships that were developed. This finding reflects those from other groups where collaboration, building relationships, and working on issues of personal interest are key for people with DS.^22^

Our program is limited in a few important ways. We did not use standardized measures to assess level of partnership or impact of collaboration on researcher perspectives. We also acknowledge that an advisory group can only provide feedback from one perspective, and it is critical that studies include collecting and understanding participant and study partner experience for ongoing studies, including the experience of individuals who are not eligible for studies. We also do not have appropriate metrics for accessibility and inclusivity of DS studies, which should be a priority for future work. We have not, to date, offered formal research training for our self‐advocates and care partners; programs like “Research Ethics for All” may be helpful to build the confidence and capacity of group members.^23^

## 5 CONCLUSION

The ACTC‐DS and ABC‐DS Research Partnership Group represents a turning point in AD research for individuals with DS. By centering the voices of self‐advocates and families, we have redefined what inclusive, ethical, and scientifically rigorous research can look like. This collaborative model not only builds trust but also ensures that research priorities reflect the lived realities of those most affected. Through this partnership, studies are becoming more accessible and relevant, with community input shaping all steps of the process from study design to outcome measures. This approach strengthens the scientific foundation while honoring the dignity and autonomy of participants. It offers a replicable framework for future research efforts seeking to align innovation with the values and needs of the communities they aim to serve. Our commitment is ongoing. We will continue working to improve the lives of people with DS by empowering self‐advocates as equal partners in research—ensuring their voices are not only heard but drive the future of discovery.

## CONFLICT OF INTEREST STATEMENT

Sarah Walter receives funding from a grant for the Alzheimer's Clinical Trials Consortium (ACTC) National Institute on Aging (NIA), National Institutes of Health (NIH: U24AG057437) paid to her institution. Lauren Ptomey receives grants from the NIA (NIH: U01HD116477, NIH R21HD111917, R33 AG078967, R01DK137986, U19 AG068054, and R01 AG063909) paid to her institution. Ann Cohen receives grant support from the NIA (NIH: U01AG051406 and U01AG051412) paid to her institution. Elizabeth Head serves as a Section Co‐Editor for Alzheimer & Dementia journal. Michael S. Rafii receives grants from NIA (NIH: R61AG066543 and R33AG066543) and contracts from Eisai and Eli Lilly, paid to his institution. He has received consulting fees from AC Immune and Ionis Pharmaceuticals. He has participated on a data safety monitoring board or advisory board for Alnylam Pharmaceuticals, Alzheon, Aptah Bio, Biohaven, Embic, Helicon, Prescient Imaging, Positrigo, and Recall Therapeutics. The other authors are members of the research partnership group and receive compensation for their time as part of a research protocol under the University of Southern California Institutional Review Board. Author disclosures are available in the supporting information.

## CONSENT STATEMENT

Consent was not required for this study.

## Supporting information



Supporting Information

Supporting Information
