# Supplementary material for: “Being brave, being seen, and having your voice heard”: Perspectives of self‐advocates and families toward accessible and impactful research of Alzheimer's disease in down syndrome
Source: Alzheimers Dement. 2025 Dec 19;21(12):e70999. doi: 10.1002/alz.70999 (PMC12715699; doi:10.1002/alz.70999)
Supplement: Supplementary file 2 — Supporting Information [file ALZ-21-e70999-s001.pdf]

“BEING BRAVE, BEING SEEN AND HAVING YOUR VOICE HEARD”

How to improve research in Alzheimer’s Disease and Down syndrome

People with Down syndrome carry a high risk for Alzheimer’s disease. Most get it by age 55. After 35, it is the leading cause of death in this population.

Most Alzheimer’s disease studies exclude people with Down syndrome. More inclusive and accessible research is needed for this population.

To help address this urgent need, a research partnership group supported by the Alzheimer’s Clinical Trials Consortium Down syndrome (ACTC-DS) and Alzheimer’s Biomarker Consortium Down syndrome (ABC-DS) was created to support research in this area.

Our group includes self-advocates, care partners, and researchers from across the USA. Together, we created recommendations for researchers and feedback to guide the design of respectful, accessible, and inclusive studies for people with Down syndrome.

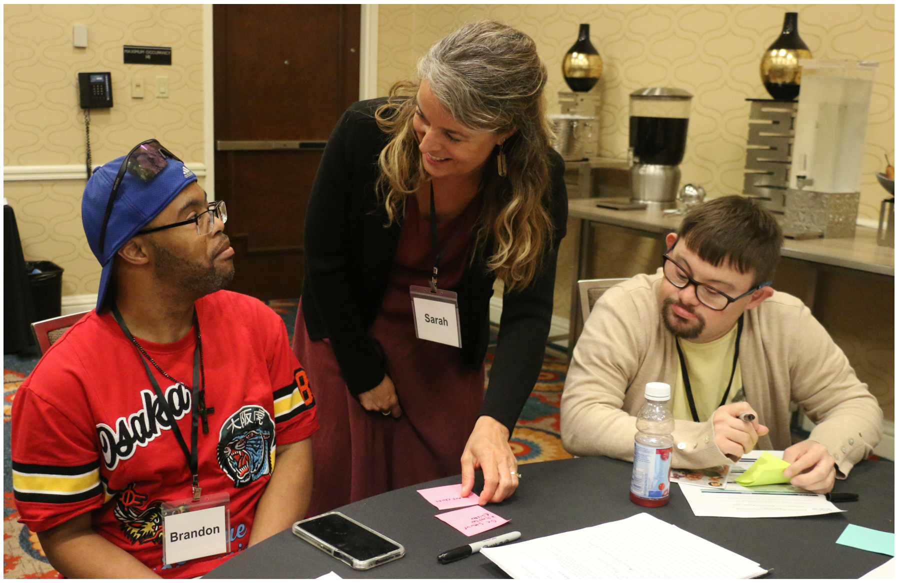

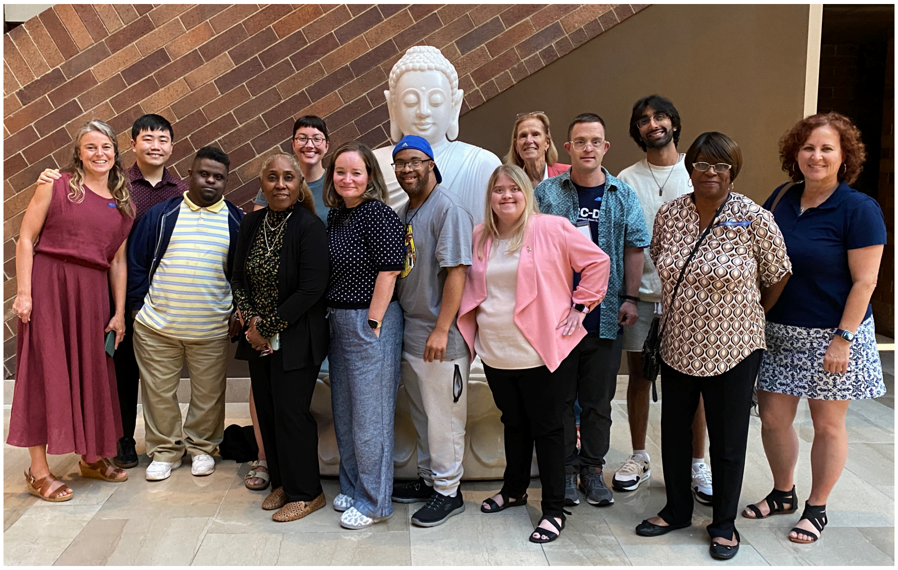

“Being Brave, Being Seen, and Having Your Voice Heard”: Perspectives of Self-Advocates and Families Towards Accessible and Impactful Research of Alzheimer Disease in Down syndrome

Sarah Walter, MSc, Lauren Ptomey, Elizabeth Head, Annie Cohen, Joseph Mike Briones, Henry Shaw, Brandon Carter, Jessica Kishner, Willie Pestolesi, Anthony Sciullo, Katy Kolb Olmstead, Aurelia Carter, Sara Kishner, Amy Kolb Tucker, Lissa Pestolesi, Maria Briones, Dana Sciullo, Kay Sciullo, Pam Shaw, Perry Chen, Michael S. Rafii

Published in
Alzheimer’s and Dementia, 2025

For questions, please contact:
Sarah Walter, MSc
ACTC Program Administrator
waltersa@usc.edu

ABC-DS
Alzheimer Biomarker Consortium-Down Syndrome

ACTC-DS
Alzheimer's Clinical Trials Consortium
Down Syndrome

5 KEY THEMES

1. Motivation

Self-advocates value their independence and are highly motivated to work with researchers alongside their care partners.

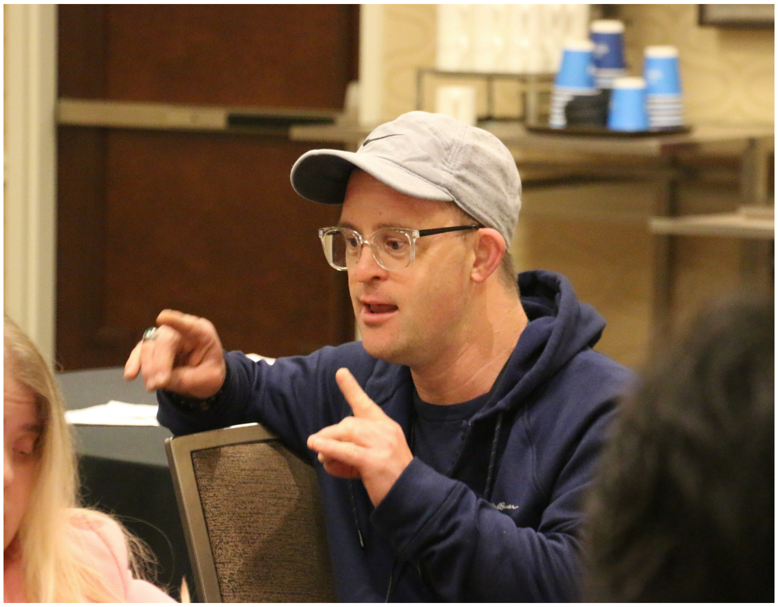

2. Respect for Self-Advocates

Participants and care partners in our group want researchers and medical staff to show respect to self-advocates.

3. Balancing Risk

Self-advocates and families weigh health needs and future dementia risk when thinking about study side effects.

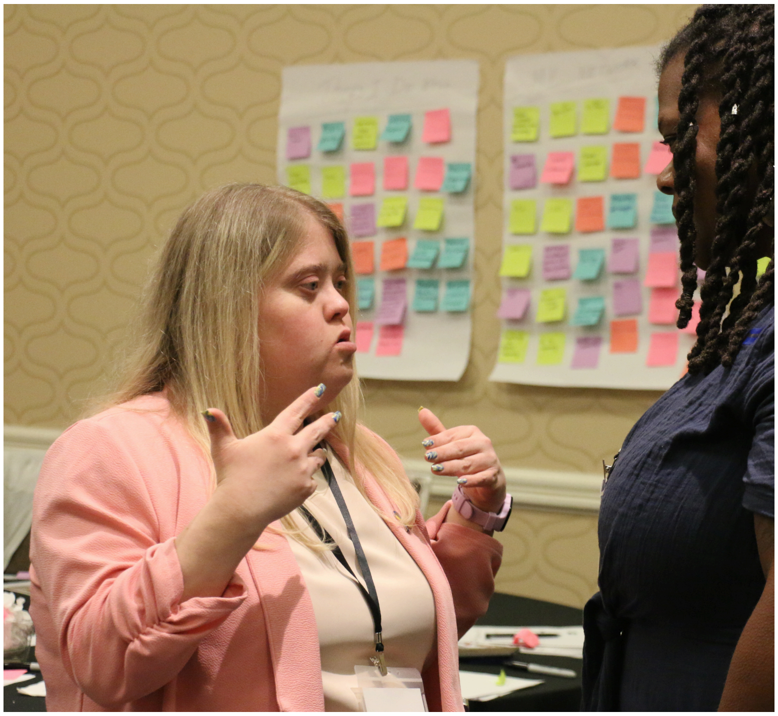

4. Information & Specialty Care

Self-advocates and care partners seek and share information to make smart choices and lower dementia risks.

5. The Value of Peer Support

Our group has served as a space for self-advocates to build and strengthen relationships with peers.

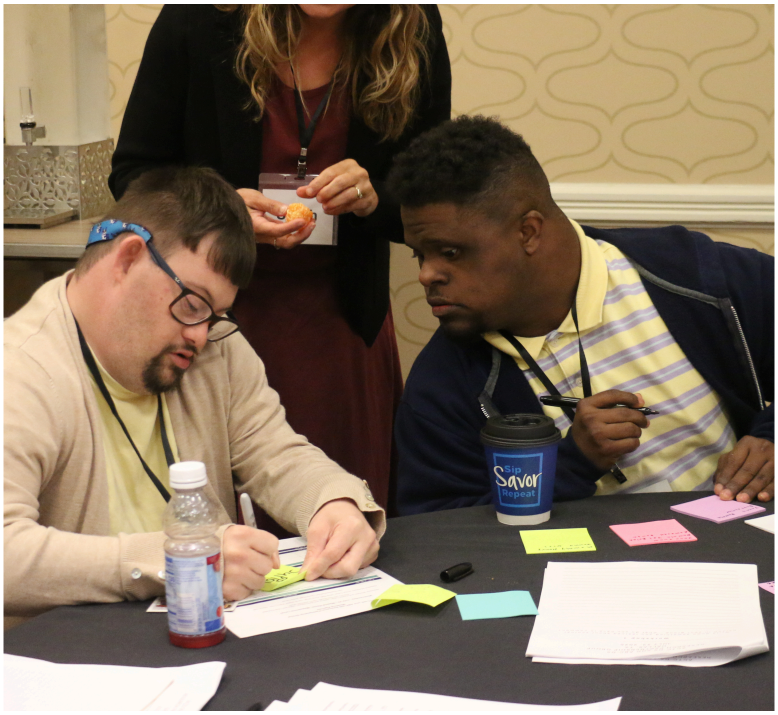

Our Recommendations for Researchers

1. **We need support and connection**
   Researchers can support all volunteers, even those who do not qualify for their study.

2. **We want to receive our research results**
   Offering results encourages people to join trials, stay involved, and feel empowered about brain health.

3. **We need information explained to us**
   Define all scientific terms. Explain how researchers support and protect participants.

4. **We need research to fit our busy lives**
   Accommodations like transportation, in-home services, etc. can help minimize disruptions.

5. **We need respectful communication**
   Train research staff to speak directly to self-advocates. Adapt materials to let them take active roles in studies.

6. **We need support during research**
   Offer breaks, activities, and learn what bothers them. Give warnings and relief if pain/discomfort may occur.

7. **We want accessible, inclusive materials**
   Advisory boards of self-advocates and families can review materials to ensure participants can read them.

8. **We want to stay connected**
   Plan to stay in regular contact with participants and those who didn't qualify or haven't joined a study yet.

9. **We want a true partnership**
   Hiring self-advocates as research staff can help those who cannot join studies and provide peer support.

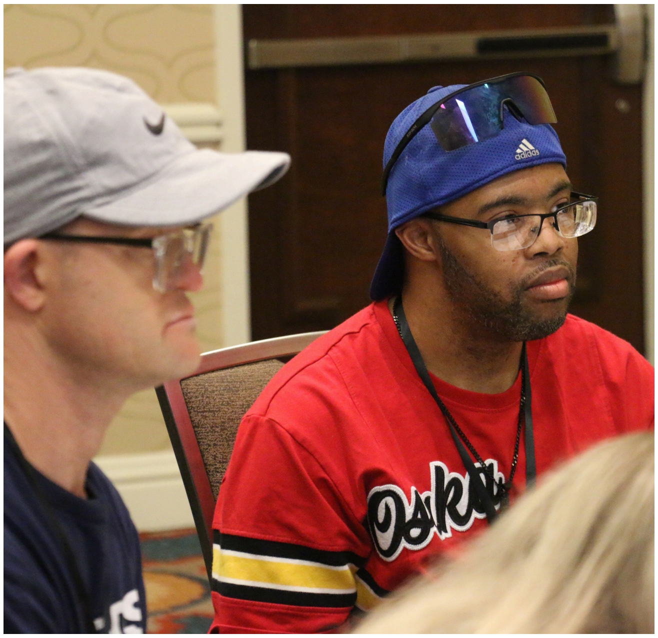

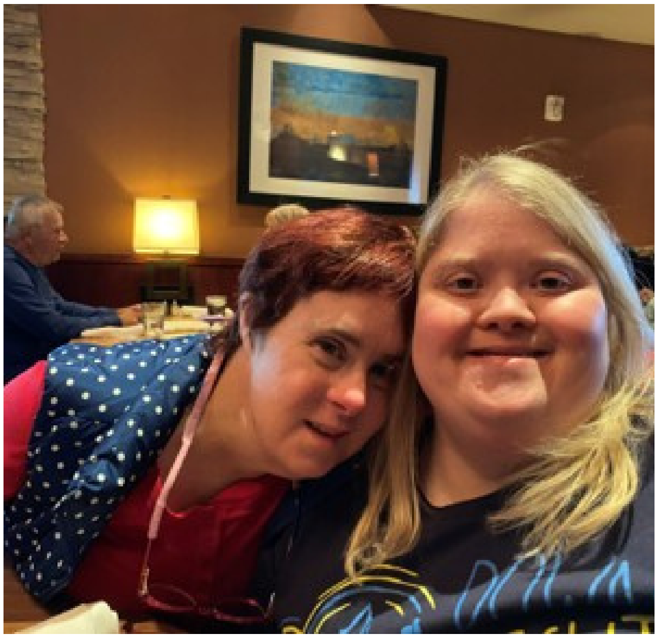

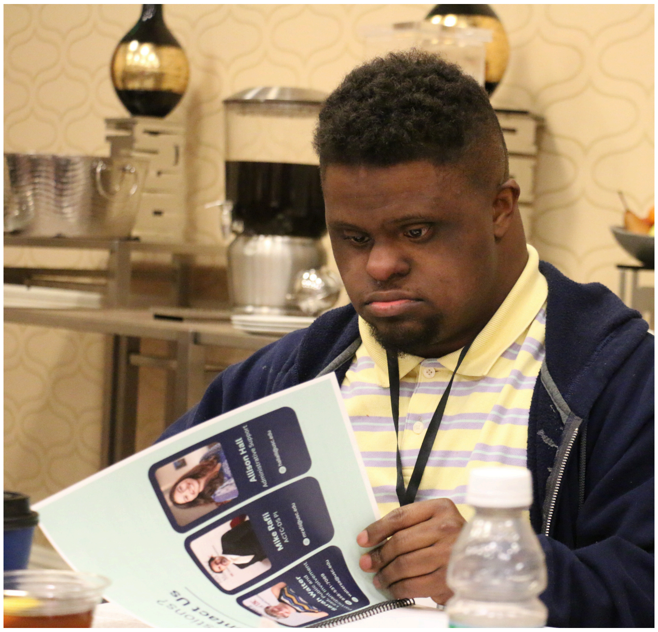

“BEING BRAVE, BEING SEEN AND HAVING YOUR VOICE HEARD”

Feedback from self-advocates to improve Alzheimer’s research

Some people with Down syndrome have a lot of trouble remembering things. They find it very hard to talk or think clearly. This is called dementia.

The most common kind of dementia is Alzheimer's disease. Most people with Down syndrome get Alzheimer’s when they grow old.

Researchers run studies about Alzheimer’s to learn how to treat it. We need more of these studies for people with Down syndrome.

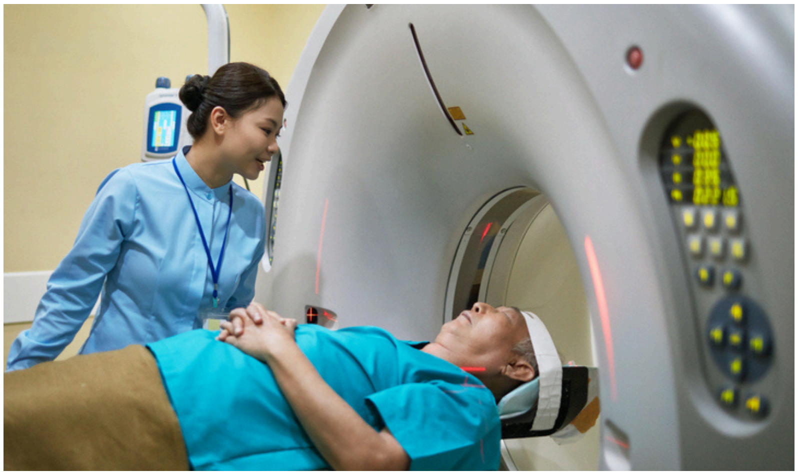

We built a team to help researchers. People with Down syndrome are part of our team. Their families are too. Our team helps improve Alzheimer’s studies for people with Down syndrome.

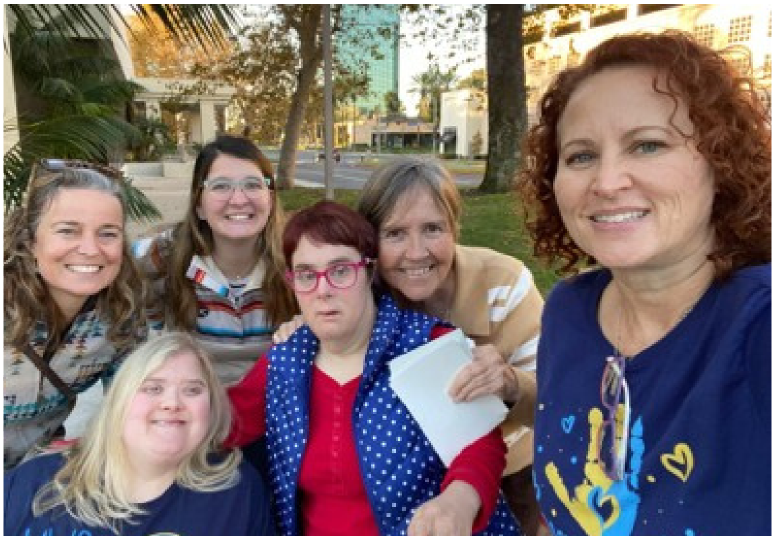

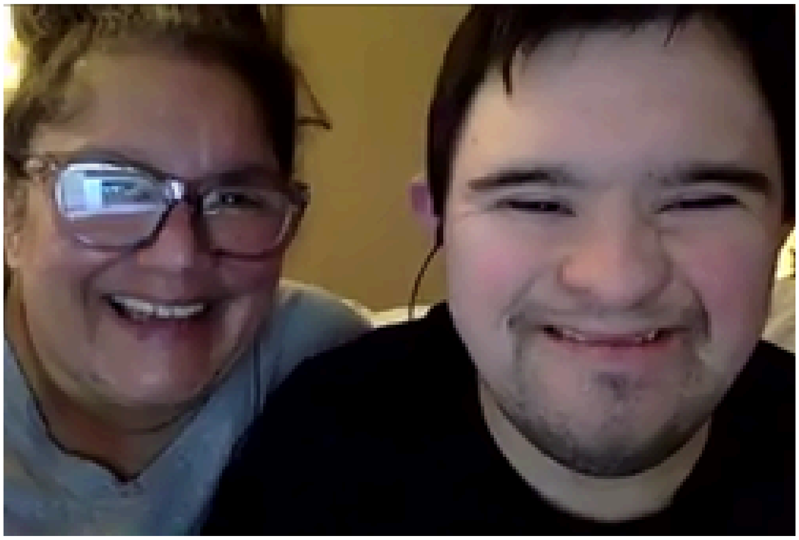

Our Advice for Researchers

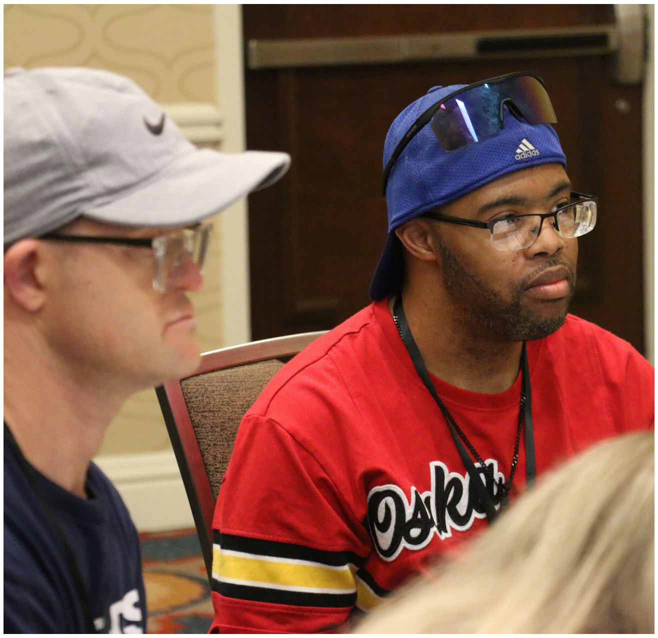

1 We need support and connection

Researchers can support all volunteers, even those who do not take part in studies.

2 We want to receive our research results

Sharing results helps us want to be involved and gives us important information about our brain health. .

3 We need information explained to us

Take extra time to explain special words and how we will be supported and feel safe.

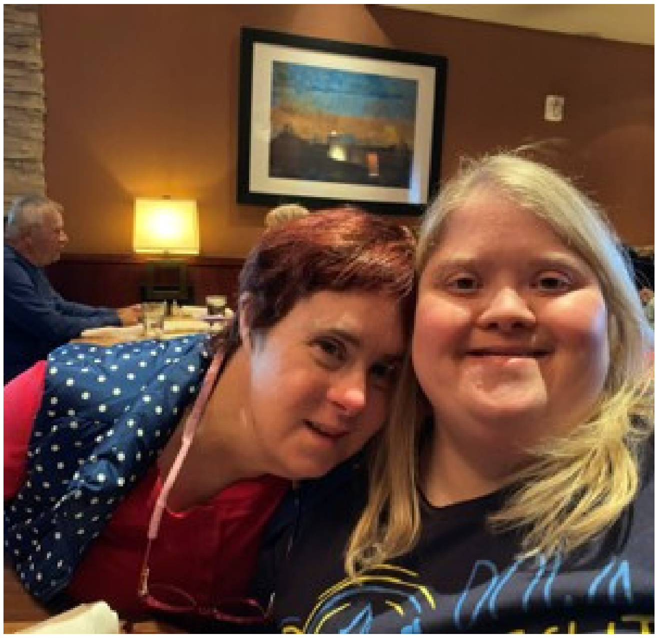

4 We need research to fit in our busy lives

Our jobs and activities are important to us! Free rides and studies from home will help.

5 We need respectful communication

Train research staff to speak directly to self-advocates. Write materials so we can understand our choices.

6 We need support during research

Offer breaks, activities, and talk about what bothers us, so we can be ready.

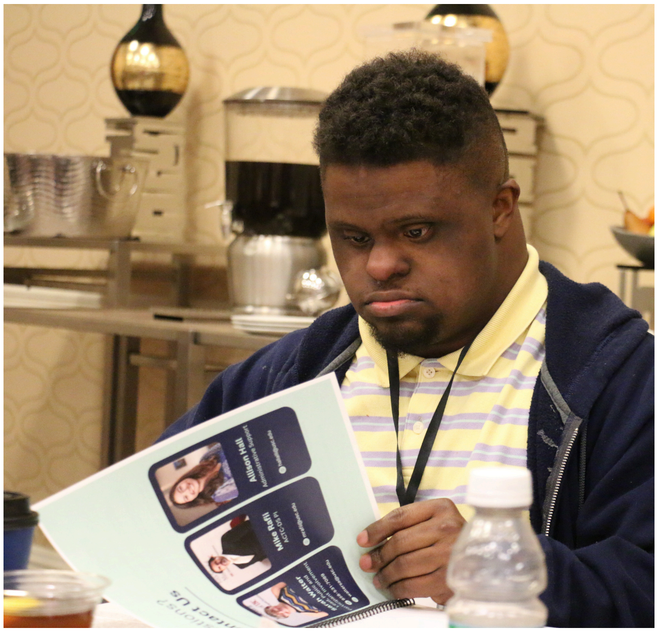

7 We want accessible, inclusive materials

Diverse pictures of people with Down syndrome shows that research is for everyone!

8 We want to stay connected

Stay in regular contact with self-advocates, families, and people that aren't taking part in research.

9 We want a true partnership

Some self-advocates might want to work as research staff and study buddies, or partner in other ways.

Why should people with Down syndrome help with research?

Not all researchers know what it's like to live with Down syndrome. Self-advocates and their families are the experts!

Our team was made so we can share feedback with researchers. We have ideas for how to make studies better for everyone.

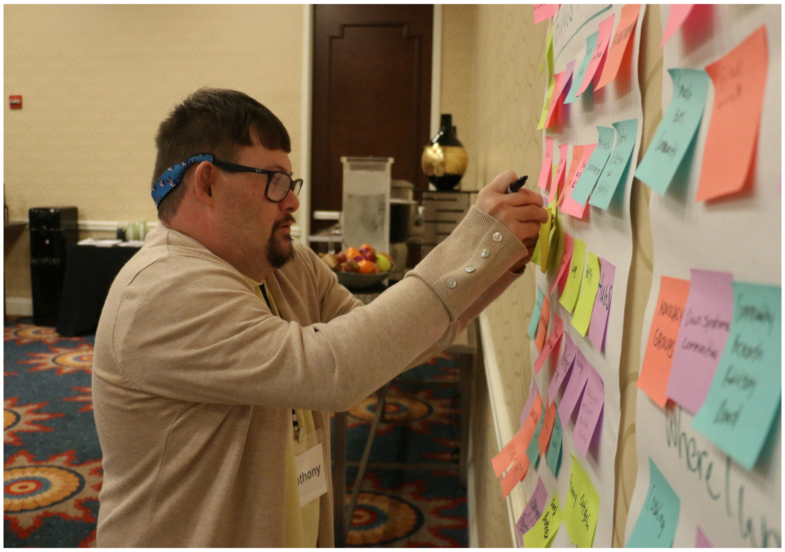

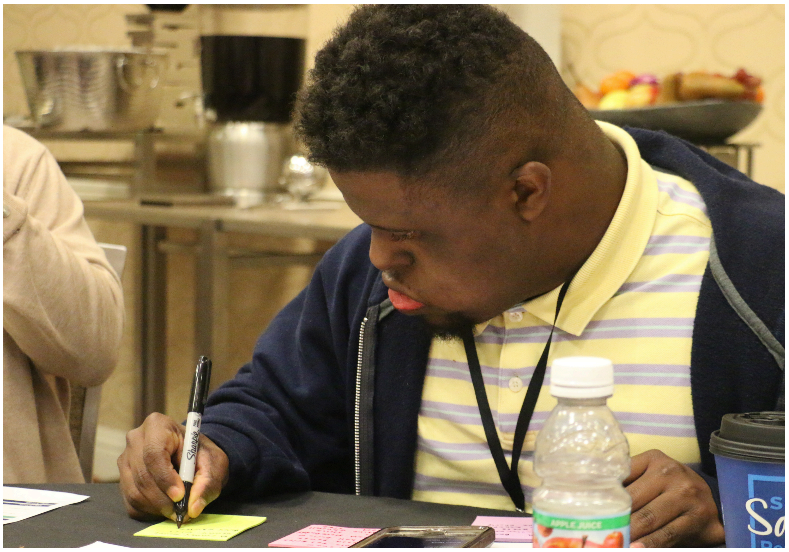

What can I do to help with research?

Studies help us learn how to keep your brain healthy. Researchers need people with Down syndrome in their studies. You can help by joining a research study!

Researchers need feedback, too. Talk to your researcher. Your feedback can help them. Ask them if they need help in other ways.

“Being Brave, Being Seen, and Having Your Voice Heard”: Perspectives of Self-Advocates and Families Towards Accessible and Impactful Research of Alzheimer Disease in Down syndrome

Sarah Walter, MSc, Lauren Ptomey, Elizabeth Head, Annie Cohen, Joseph Mike Briones, Henry Shaw, Brandon Carter, Jessica Kishner, Willie Pestolesi, Anthony Sciullo, Katy Kolb Olmstead, Aurelia Carter, Sara Kishner, Amy Kolb Tucker, Lissa Pestolesi, Maria Briones, Dana Sciullo, Kay Sciullo, Pam Shaw, Perry Chen, Michael S. Rafii

Published in
Alzheimer’s and Dementia, 2025

For questions, please contact:
Sarah Walter, MSc
ACTC Program Administrator
waltersa@usc.edu

ABC-DS
Alzheimer Biomarker Consortium-Down Syndrome

ACTC-DS
Alzheimer's Clinical Trials Consortium
Down Syndrome
